# Relaxin-2 as a Potential Biomarker in Cardiovascular Diseases

**DOI:** 10.3390/jpm12071021

**Published:** 2022-06-21

**Authors:** Alana Aragón-Herrera, Sandra Feijóo-Bandín, Laura Anido-Varela, Sandra Moraña-Fernández, Esther Roselló-Lletí, Manuel Portolés, Estefanía Tarazón, Oreste Gualillo, José Ramón González-Juanatey, Francisca Lago

**Affiliations:** 1 Cellular and Molecular Cardiology Unit and Department of Cardiology, Institute of Biomedical Research of Santiago de Compostela (IDIS-SERGAS), Travesía da Choupana s/n, 15706 Santiago de Compostela, Spain; sandra.feijoo.bandin@sergas.es (S.F.-B.); laura.anido.varela@sergas.es (L.A.-V.); sandra.morana.fernandez@usc.es (S.M.-F.); jose.ramon.gonzalez.juanatey@sergas.es (J.R.G.-J.); francisca.lago.paz@sergas.es (F.L.); 2 Network Research Centre for Cardiovascular Diseases (CIBERCV), Institute of Health Carlos III, C/Monforte de Lemos 3-5, Pabellón 11, Planta 0, 28029 Madrid, Spain; esther_rosello@iislafe.es (E.R.-L.); portoles_man@gva.es (M.P.); estefania_tarazon@iislafe.es (E.T.); 3 Cardiology Group, Center for Research in Molecular Medicine and Chronic Diseases (CIMUS), University of Santiago de Compostela and Health Research Institute, University Clinical Hospital of Santiago de Compostela, Avenida Barcelona s/n, 15782 Santiago de Compostela, Spain; 4 Cardiocirculatory Unit, Health Institute La Fe University Hospital (IIS La Fe), Avda. de Fernando Abril Martorell 106, 46026 Valencia, Spain; 5 Laboratory of Neuroendocrine Interactions in Rheumatology and Inflammatory Diseases, Institute of Biomedical Research (IDIS) and Xerencia de Xestión Integrada de Santiago de Compostela (XXIS/SERGAS), Travesía da Choupana s/n, 15706 Santiago de Compostela, Spain; oreste.gualillo@sergas.es

**Keywords:** relaxin-2, relaxin, biomarker, cardiovascular disease, cardiac, heart

## Abstract

The pleiotropic hormone relaxin-2 plays a pivotal role in the physiology and pathology of the cardiovascular system. Relaxin-2 exerts relevant regulatory functions in cardiovascular tissues through the specific receptor relaxin family peptide receptor 1 (RXFP1) in the regulation of cardiac metabolism; the induction of vasodilatation; the reversion of fibrosis and hypertrophy; the reduction of inflammation, oxidative stress, and apoptosis; and the stimulation of angiogenesis, with inotropic and chronotropic effects as well. Recent preclinical and clinical outcomes have encouraged the potential use of relaxin-2 (or its recombinant form, known as serelaxin) as a therapeutic strategy during cardiac injury and/or in patients suffering from different cardiovascular disarrangements, especially heart failure. Furthermore, relaxin-2 has been proposed as a promising biomarker of cardiovascular health and disease. In this review, we emphasize the relevance of the endogenous hormone relaxin-2 as a useful diagnostic biomarker in different backgrounds of cardiovascular pathology, such as heart failure, atrial fibrillation, myocardial infarction, ischemic heart disease, aortic valve disease, hypertension, and atherosclerosis, which could be relevant in daily clinical practice and could contribute to comprehending the specific role of relaxin-2 in cardiovascular diseases.

## 1. Introduction

Despite significant advances in cardiovascular medicine, cardiovascular diseases (CVDs) continue to be the leading cause of morbidity and mortality in modern society worldwide, taking an estimated 17.9 million lives each year, and they remain a critical health concern globally [1]. The early identification of the onset of CVDs could substantially improve the prognosis and management of these pathologies; however, cardiac troponins and natriuretic peptides, the most famous and clinically applied diagnostic biomarkers for the management of CVDs, present numerous limitations [2,3]. For example, the high-sensitivity assays for cardiac troponins I and T counteract the insufficient diagnostic specificity for acute myocardial infarction (MI), while plasmatic levels of the B-type natriuretic peptides are usually confounded by age or non-CVD problems like renal dysfunction [4]. This enforces an imperious necessity for additional specific and improved diagnostic biomarkers with high predictive value, which can solve current significant flaws, improve diagnostic accuracy, and provide early risk stratification in CVDs during daily clinical practice.

Relaxin-2 is a natural peptide hormone that was first discovered in 1926 by Frederick Hisaw after his observations in guinea pigs and gophers, in which relaxin-2 induced the relaxation of the pubic ligaments and the softening of the pubic symphysis to facilitate delivery in pregnant animals [5]. Several years later, in the 1950s, relaxin-2 began to be investigated clinically as a partially purified extract of porcine ovaries, and its broad endocrine role during female reproduction was clearly established [6,7]. In the successive decades, vast advances have been achieved in basic and clinical relaxin-2 research in male and female mammals, including the ability to synthesize highly purified recombinant human relaxin-2, called serelaxin [8,9,10]. Even though human relaxin-2 was initially discovered as a hormone principally secreted by the corpus luteum of the ovary, which modulates the adaptive changes in pregnancy and reproductive function (including relaxation of the birth canal, decidualization, embryo implantation, suppression of uterine contractility, increase in maternal renal plasma flow, glomerular filtration and cardiac output, and participation in nipple development) [11], it is now widely known that relaxin-2 is also synthesized in different tissues of non-pregnant women and in men; indeed, it is currently considered one of the most pleiotropic hormones of the human body, capable of inducing a wide range of actions besides those of reproduction [12]. The availability of serelaxin has permitted the study of relaxin-2’s effects in cardiovascular, renal, hepatic, and brain tissues, as well as its evaluation in several randomized placebo-controlled clinical trials [13,14]. To date, the pharmacology and specific mechanisms of the systemic action of treatment with serelaxin have been studied in more than 15 human clinical trials, including in healthy subjects [9,15,16] and in pregnancy [17,18], preeclampsia [19] (clinicaltrials.gov identifiers NCT00333307 and NCT01566630), acute and chronic heart failure (HF) [20,21,22,23,24,25,26], systemic sclerosis [27,28,29], renal and hepatic impairment [30,31], and cirrhosis [32]. 

**Figure 1:**
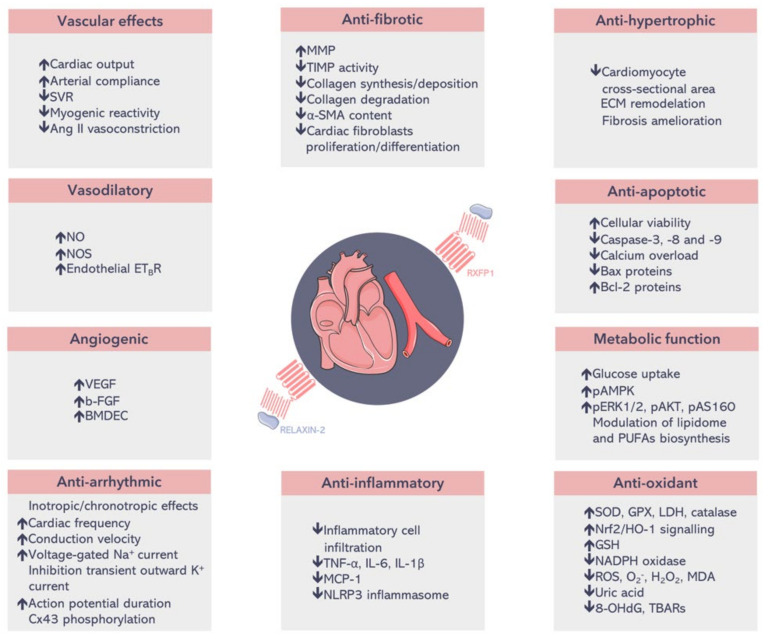
Relaxin-2’s beneficial effects on the heart and the vasculature. Through its cognate receptor relaxin family peptide receptor 1 (RXFP1), relaxin-2 is able to induce a myriad of cardioprotective roles [33,34,39,40]. α-SMA: α-smooth muscle actin. 8-OHdG: 8-hydroxy-2-deoxyguanosine. b-FGF: basic-fibroblast growth factor. BMDEC: bone marrow-derived endothelial cells. Cx43: connexin43. ECM: extracellular matrix. ET_B_R: endothelin B receptor. GPX: glutathione peroxidase. GSH: glutathione. IL-1β: interleukin-1β. IL-6: interleukin-6. LDH: lactate dehydrogenase. MCP-1: monocyte chemoattractant protein-1. MDA: malondialdehyde. MMP: matrix metalloproteinase. NADPH: nicotinamide-adenine-dinucleotide phosphate. NLRP3: nucleotide-binding oligomerization domain, leucine-rich repeat and pyrin domains-containing protein 3. NO: nitric oxide. NOS: nitric oxide synthase. Nrf2/HO-1: nuclear factor erythroid 2-related factor transcription factor/hemoxygenase 1. pAMPK: phospho-adenosine monophosphate-activated protein kinase. pAS160: phospho-Akt substrate of 160 kDa. pERK1/2: phospho-extracellular signal-regulated protein kinases 1 and 2. PUFAs: polyunsaturated fatty acids. ROS: reactive oxygen species. RXFP1: relaxin family peptide receptor 1. SOD: superoxide dismutase. SVR: systemic vascular resistance. TBARs: thiobarbituric acid-reactive substance. TIMP: tissue inhibitor of metalloproteinase. TNF-α: tumour necrosis factor-α. VEGF: vascular endothelial growth factor. ↑: increase. ↓: decrease. Parts of the figure were drawn by using pictures from Servier Medical Art. Servier Medical Art by Servier is licensed under a Creative Commons Attribution 3.0 Unported License (https://creativecommons.org/licenses/by/3.0/ (accessed on 1 May 2022)).

Endogenous relaxin-2 has been closely associated with a myriad of cardioprotective effects in the heart and blood vessels and has been proposed as a therapeutic strategy for several CVDs such as HF, atrial fibrillation (AF), acute myocardial infarction (AMI), ischemic heart disease, and hypertension [33]. Through the activation of its cognate receptor relaxin family peptide receptor 1 (RXFP1) and the downstream of several molecular signalling pathways in the cardiovascular system, relaxin-2 is able to induce vasodilatation and angiogenesis; increase arterial compliance and cardiac output; reduce vascular resistance; exert anti-fibrotic roles by the regulation of extracellular matrix (ECM) turnover and remodelling; reduce inflammation, apoptosis, and oxidative stress; induce chronotropic and inotropic effects; and suppress ventricular and atrial arrhythmias by countering electrical remodelling (Figure 1) [33,34]. Relaxin-2 promotes the growth and maduration of cardiomyocytes and avoids their apoptosis, acts as a cardiostimulant through the increase in the calcium sensitivity of cardiac myofilaments and myofilament phosphorylation by protein kinase C, and protects cardiomyocytes from hypoxia/reoxygenation injury by upregulating neurogenic locus notch homolog protein 1 (Notch-1) signalling [35,36,37,38]. Moreover, relaxin-2 can also regulate cardiomyocyte energetic metabolism through the activation of adenosine monophosphate-activated protein kinase (AMPK), extracellular signal-regulated kinase 1/2 (ERK1/2), protein kinase B (Akt), and the Akt substrate of 160 kDa (AS160) (key sensors and regulators of cellular energy homeostasis) as well as the stimulation of glucose uptake in cardiomyocytes; it is also able to modulate the lipidome and the endogenous synthesis of long-chain polyunsaturated fatty acids (PUFAs) at cardiac level in rats (Figure 1) [39,40]. Additionally, relaxin-2 regulates cardiac fibroblast proliferation, inhibits the differentiation of transforming growth factor-β1 (TGF-β1)/Smad3-induced cardiac fibroblasts towards myofibroblasts, decreases collagen synthesis and stimulates ECM degradation through increases in the matrix metalloproteinase-2 (MMP-2)/tissue inhibitor of metalloproteinase-2 (TIMP-2) ratio and the secretion of interleukin-10 (IL-10) in cardiac fibroblasts [41,42,43]. In aortic endothelial cells exposed to vascular damage and dysfunction, relaxin-2 activates Akt, up-regulates the gene expression of pyruvate dehydrogenase kinase isoform 4 (PDK4), represses tumour necrosis factor (TNF)-induced nuclear factor-kB and activator protein, counterbalances apoptosis and oxidative stress, modulates endothelial nitric oxide synthase (eNOS) expression and ameliorates vasodilator prostacyclin production [44,45,46]. These outcomes suggest that relaxin-2 improves endothelial dysfunction and functions as a natural and potent therapeutic vasoprotector. Owing to this preclinical evidence regarding the cardioprotective effect of relaxin-2, researchers and pharmaceutical companies became interested in the potential of this hormone as a new therapy for human CVDs. In fact, the phase II Preliminary study of RELAXin in Acute Heart Failure (Pre-RELAX-AHF) and the phase III RELAX-AHF clinical trials showed that intravenous treatment with serelaxin could relieve some symptoms and signs and decrease mortality in patients with acute HF (AHF) [23,24], although the larger RELAX-AHF-2 study failed in replicating the clinical endpoints observed in previous clinical trials [47,48]. Recently, researchers have increased their interest in the development of serelaxin mimetics such as low-molecular-weight oligopeptide analogues (for example, B7-33) and nonpeptidic agonists (like ML290) and in the design of nanoparticles systems (for example, conjugating relaxin-2 to superparamagnetic iron oxide nanoparticles (RLX-SPION) or using nanoparticles for relaxin-2 gene therapy), which could improve the pharmacological and pharmacokinetic features of serelaxin and solve the problems associated with its administration in humans [49,50,51,52].

Owing to its remarkable role during reproduction and pregnancy, relaxin-2 has been suggested as a biomarker in different gynaecological and obstetrical settings such as preterm delivery, preeclampsia, ectopic pregnancies, and even ovarian cancer [53,54,55,56,57,58,59]. Apart from pregnancy, serum and plasma relaxin-2 levels could be associated with CVDs, a fact that in parallel with the role of relaxin-2 during renal and systemic haemodynamic adaptations in pregnancy, and its clear beneficial effect on cardiac function, has prompted the interest in relaxin-2 as an endogenous biomarker of several CVDs [34]. The hypothesis that relaxin-2 could be a promising physiological marker involved in the natural protection against cardiovascular disarrangements of women during fertile life has encouraged researchers’ interest in the utility of relaxin-2 as a predictor of cardiovascular pathology in different population groups of both sexes [60]. In this review, we will emphasize the relevance of assaying relaxin-2 levels as a useful diagnostic and prognostic biomarker in different backgrounds of CVDs, which could be relevant for daily clinical practice in the management of these syndromes.

## 2. Endogenous Levels of Relaxin-2 in Physiological Conditions

Circulant human relaxin-2 is mainly produced and secreted by the corpus luteum in the ovary during the menstrual cycle and during pregnancy, which explains its principal biological function during reproduction and gestation [11,61]. Relaxin-2 is released to peripheral circulation during the luteal phase of the menstrual cycle, in which its levels in plasma or serum are low/moderate, but then augment on days 9–12 post-ovulation, reaching a small peak of concentration (50–100 pg/mL) in the late luteal phase of the cycle (Table 1) [62,63,64,65]. After conception, relaxin-2 plasma levels increase drastically and during the different periods of gestation could reach peaks of concentration of 1000–2500 pg/mL in the first trimester; these levels slightly decrease throughout the second trimester and remain stable throughout the rest of the pregnancy with a concentration of approximately 500 pg/mL (Table 1) [66,67,68]. At the end of pregnancy and in postpartum, relaxin-2 levels decline quickly, reaching normal non-pregnant levels in the second postpartum week (Table 1) [66,67,69]. Intriguingly, relaxin-2 levels drastically increased in superovulated singleton pregnancies and in multifetal pregnancies, an observation that could be explained by the presence of multiple corpus luteum together with a greater function of this structure (Table 1) [66].

In healthy men and women, and during menopause, circulant levels of relaxin-2 range from 10 to 50 pg/mL but could even be undetectable (Table 1) [67,70,71]. In men, the prostate gland shows higher relaxin-2 levels, which are probably secreted to the circulation and seminal plasma [72]. Curiously, athletic women treated with oral contraceptives (OC) have shown lower serum relaxin-2 levels, which could possibly be because this therapy suppresses ovulation and the formation of the corpus luteum, an observation that agrees with the fact that amenorrhoeic athletic women have lower levels of relaxin-2 in serum [73,74]. However, these results do not support the previous observations of Wreje et al. (1995), who found that serum levels of relaxin-2 were increased during the use of OC in healthy women, possibly due to the existence of sources for relaxin-2 production other than the corpus luteum in the non-pregnant woman [75]. Moreover, it might be that the presence of ethinyloestradiol in OC, in a similar manner to oestradiol, has a direct effect and could induce changes in the secretion of relaxin-2 [75,76,77].

As we have mentioned above, relaxin-2 levels could be potentially employed as a circulant gynaecological and obstetrical physiopathological biomarker owing to its traditional role in reproduction [53,54,55,56,57,58,59] and also in premenstrual disorders such as premenstrual dysphoria [78]. Notwithstanding its documented role in the reproductive system, relaxin-2 is also synthesized in different organs and tissues of the human anatomy such as the brain, lungs, heart, blood vessels, liver, pancreas, small intestine, kidney, and bladder, and it is released into the circulation [12,79]; it is hypothesized that changes in the circulatory levels of relaxin-2 could be related to different pathological settings like diabetes [80], cancer [81,82], orthopaedical disarrangements [83,84,85], systemic and multiple sclerosis [71,86], end-stage kidney disease [87], and of course, CVDs, as we are going to explain in the following section.

**Table 1:** Relaxin-2 plasma or serum levels in different physiological conditions.

Condition	Subjects (n)	Mean Relaxin-2 Concentration	Main Results	Reference
Healthy men	Men (1)	91.5 ± 13.8 ng/mL	Relaxin-2 concentrations ↓ in the post-menopausal and male subjects in other conditions	[88]
Menstrual cycle	Periovulatory (1)	142.9 ± 17.4 ng/mL		
	Follicular phase (1)	112.8 ± 24.9 ng/mL		
Pregnancy	Pregnant (1)	128.7 ± 19.1 ng/mL		
Menopause	Post-menopausal (1)	46.5 ± 7.5 ng/mL		
Spontaneously pregnancy	5 weeks (4)	315 (20–1200) pg/mL	Relaxin-2 levels ↑ during different weeks of pregnancy but start to ↓ in week 20	[66]
	7 weeks (9)	923 (230–1920) pg/mL		
	11 weeks (20)	1294 (538–3480) pg/mL		
	14 weeks (18)	1122 (400–2430) pg/mL		
	20 weeks (19)	555 (117–1712) pg/mL		
	26 weeks (35)	515 (134–1808) pg/mL		
	30 weeks (30)	568 (255–1774) pg/mL		
	38 weeks (25)	494 (212–1930) pg/mL		
Pregnancy with superovulation	Singleton pregnancy (15)	179–14,633 pg/mL	Relaxin-2 levels increase with conceptus number	[66]
	Twin pregnancy (14)	223–13,750 pg/mL		
	Triplet pregnancy (28)	850–21,700 pg/mL		
	Quadruplet pregnancy (10)	1030–18,700 pg/mL		
	Quintuplet pregnancy (10)	4820–28,800 pg/mL		
Women with normal ovarian function	Basal follicular (8)	13 ± 5.0 pg/mL *	Relaxin-2 levels in the luteal phase were significantly ↑	[64]
	Luteal (8)	41 ± 11 pg/mL *		
Healthy men	Men (9)	8.3 ± 0.8 pg/mL *	Relaxin-2 levels were ↑ in women compared with men	[71]
Healthy women	Women in reproductive age (18)	15.3 ± 1.3 pg/mL *		
	Menopausal women (23)	14.9 ± 0.9 pg/mL *		
Menstrual cycle	Days 1–4 (20)	82.1 ± 23.8 pg/mL *	Relaxin-2 were ↑ in the luteal phase	[65]
	Days 12–14 (20)	86.2 ± 25.5 pg/mL *		
	Days 20–23 (20)	94.1 ± 27.1 pg/mL *		
Pregnancy	Prepregnancy (13)	52.88 ± 29.66 pg/mL	Relaxin-2 levels at each time point during pregnancy were ↑ except at 26 weeks of pregnancy Relaxin-2 ↓ at 36 weeks’ gestation and in postpartum when compared with pregnancy state	[67]
	6 weeks (12)	1949.91 ± 895.62 pg/mL		
	10 weeks (11)	1713.08 ± 516.70 pg/mL		
	16 weeks (12)	1345.82 ± 751.56 pg/mL		
	26 weeks (13)	1493.66 ± 2552.82 pg/mL		
	36 weeks (12)	859.93 ± 376.28 pg/mL		
	Postpartum (11)	53.18 ± 69.71 pg/mL		

Table 1 summarizes the most important works describing relaxin-2 circulatory levels in several physiological situations. In general, there is an increase in relaxin-2 levels in pregnancy and during the ovulation process, but there exists a huge variability in its concentration in almost all the studies reported. Relaxin-2 concentration is expressed in mean, mean ± standard error of the mean (SEM), mean ± standard deviation (SD), or range depending on the study. * Measured in serum. ↑: relaxin-2 levels increase. ↓: relaxin-2 levels decreased.

## 3. Relaxin-2 as a Biomarker in Cardiovascular Disease

There exists enough evidence supporting the striking influence of the hormone relaxin-2 on cardiovascular physiopathology in its exerting direct cardiac effects (including anti-fibrotic, anti-hypertrophic, anti-apoptotic, anti-oxidant, anti-inflammatory and anti-arrhythmic effects, as well as the modulation of cardiomyocytes’ metabolic function) and indirect cardiac actions mediated by vascular effects, as we have previously summarized [33,34]. Moreover, several reports have described that relaxin-2 is present in many biological fluids, including blood, and that the circulatory levels of relaxin-2 could be associated with the diagnosis and the prognosis of different diseases [82,89,90,91]. Despite the discrepant results described in RELAX-AHF-2 regarding the use of relaxin-2 as a therapeutical strategy, it is not reasonable to dismiss its function in the physiopathology of several CVDs given its potential role as a local mediator or to act at normal blood levels [47,92].

It is hypothesized that endogenous relaxin-2 could be a fundamental physiologic agent implicated in natural protection or compensatory mechanisms against cardiovascular pathology. In fact, the incidence of cardiovascular diseases in women is very low until the period of menopause, when it markedly augmented and reaches an incidence comparable with that in men, although with a delay of approximately 20 years [93]. This assumption may be explained by the cardioprotective effects of active substances synthesized by the corpus luteum and secreted to circulation, such as relaxin-2 [61,94]. During the last decade, various researchers have focused their studies on the analysis of relaxin-2 plasma levels in different pathological conditions in order to ascertain its promising and novel implications as a biomarker in several diseases but focusing on its role in cardiovascular pathology [60]. In the following subsections, we will focus on the most relevant studies regarding relaxin-2 circulatory levels in different backgrounds of CVDs, especially in HF, AF, ischemic heart disease, MI, aortic valve disease, hypertension, and atherosclerosis.

### 3.1. Heart Failure

**Figure 2:**
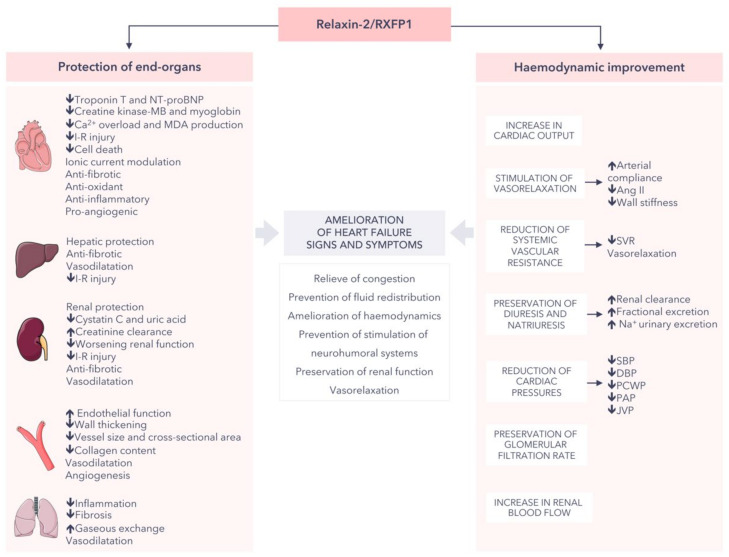
Effects mediated by relaxin-2 (or serelaxin) that counteract end-organ damage and improve haemodynamic imbalance during heart failure [12,15,33,95]. Ang II: angiotensin II. DBP: diastolic blood pressure. I-R: ischemia-reperfusion. MDA: malondialdehyde. NT-proBNP: N-terminal pro-B-type natriuretic peptide. PAP: pulmonary artery pressure. JVP: jugular venous pressure. PCWP: pulmonary capillary wedge pressure. SBP: systolic blood pressure. SVR: systemic vascular resistance. Parts of the figure were drawn by using pictures from Servier Medical Art. Servier Medical Art by Servier is licensed under a Creative Commons Attribution 3.0 Unported License (https://creativecommons.org/licenses/by/3.0/ (accessed on 1 May 2022)).

Recent preclinical and clinical works have emphasized relaxin-2 as a promising treatment of both the short- and long-term consequences of HF [33]. Endogenous relaxin-2 may counteract end-organ damage and consequently provide a long-term treatment with this hormone will benefit patients with HF via the inhibition of inflammation, fibrosis, apoptosis, and oxidative stress; the induction of angiogenesis; and the improvement of the haemodynamics (at systemic, cardiac, and renal levels), together with the relief of congestion and the stimulation of vasorelaxation (Figure 2) [12,15,33,95]. In this context, recent studies have described the promising benefit of assaying endogenous relaxin-2 levels as a biomarker for predicting disease severity in different backgrounds of HF.

Relaxin-2 is constitutively expressed in human cardiovascular tissues, and it is described that its expression is significantly increased in the failing human myocardium with augmented left ventricular filling pressure [70]. Moreover, in severe chronic heart failure (CHF) and in failing myocardium, a positive difference in relaxin-2 concentration between the aorta and coronary sinus (transcardiac relaxin-2 gradient) has suggested that the heart is a source of circulating relaxin-2 during this pathology as an attempt to compensate for deleterious conditions [70]. Conversely, negative transcardiac gradient and relaxin-2 extraction were reported during moderate CHF in patients undergoing coronary artery bypass grafting surgery but with no HF and preserved left ventricle ejection fraction (LVEF), and in non-failing hearts, which suggests that the participation of the heart in circulating relaxin-2 differs according to the presence or absence of heart disease [70,96].

In general, there is a trend towards increased levels of circulant relaxin-2 during HF. Relaxin-2 plasma levels are increased during CHF regarding healthy states (Table 2) and have shown a positive correlation with brain natriuretic peptide (BNP) and collagen I, and a negative correlation with creatinine clearance rate (Ccr) [70,97,98,99]. This increase in relaxin-2 plasma levels seems to be based on NYHA (New York Heart Association) class functional classification [98,99], and it is confirmed that relaxin-2 plasma levels and relaxin-2 gene expression in the myocardium are equivalent to the severity of HF [70], which prompted the use of endogenous relaxin-2 for a preliminary assessment of CHF severity and prognosis of the disease. In patients with HF with reduced ejection fraction (HFrEF), plasmatic relaxin-2 concentrations could be four- to six-fold higher in moderate HF (class II NYHA), and twelve- to sixteen-fold higher in severe HF (class IV NYHA), when compared with individuals without heart disease [70]. These results highlight that plasmatic relaxin-2 is related to pathophysiological events of CHF such as myocardial distension and fluid overload [70]. Moreover, it has also been suggested that relaxin-2 could predict the severity of cardiovascular events in CHF patients within 180 days after discharge [98], and the combined assessment of relaxin-2 and BNP had a remarkable potential application in clinical daily practice, improving the sensitivity and specificity of diagnosis for decompensated CHF when compared with assaying BNP alone [99]. Opposing that evidence, some authors did not find changes in circulatory relaxin-2 levels in patients with CHF (Table 2) or in the severity of the disease [100], were unable to confirm if relaxin-2 could predict HF readmission or death over the following year, and also reported no correlation between relaxin-2 and N-terminal (NT)-proBNP, a strong prognostic marker of HF [97]. In another work with patients with severe aortic valve stenosis (AVS) with and without HF, plasmatic relaxin-2 did not change in HF, and it seemed to be independent of the presence, type, or severity of HF (Table 2) [100]. Circulating relaxin-2 tended to increase in stable chronic HFrEF when compared with healthy subjects at rest or after physical exercise, although no statistical differences were observed (Table 2) [101]. In patients with HF secondary to ischemic heart disease, relaxin-2 levels were significantly reduced during the recovery after dynamic exercise and were positively correlated with cardiac power output while inversely correlated with NT-proBNP and NT-pro atrial natriuretic peptide (ANP) and with severity of HF (Table 2) [102]. In a group of women with HF resulting from peripartum cardiomyopathy (PPCM), increased serum relaxin-2 levels at admission were associated with higher LVEF at 2 months post-partum and with smaller left ventricle (LV) systolic diameter (Table 2) [90]. These findings demonstrate a faster myocardial recovery and a cardioprotective role induced by endogenous relaxin-2 during HF related to PPCM and manifest the possibility of using serelaxin as a therapeutic strategy to facilitate recovery in this syndrome [90].

During AHF, serum relaxin-2 concentration at admission was associated with pulmonary hypertension and right heart overload (Table 2) [91]. In fact, AHF patients with higher levels of relaxin-2 had a higher prevalence of right ventricle (RV) dysfunction and right chamber dilatation, showing a significantly higher sodium retention score and more pronounced peripheral oedema [91]. Patients in the upper half of relaxin-2 distribution showed significantly higher systolic pulmonary artery pressure (sPAP), right atrial (RA) and RV dilatation, higher prevalence of systolic RV dysfunction, and less inferior vena cava (IVC) diameter variability [91]. Pintalhao et al. (2017) reported an association between clinical and echocardiographic markers of right heart overload and serum relaxin-2 levels at admission, highlighting the promising use of relaxin-2 as a biomarker of RV overload during the acute setting of HF [91]. Miró et al. (2018) observed that AHF patients achieving relaxin-2 plasma levels like those described during pregnancy could survive longer [105]. When comparing pulmonary arterial hypertension (PAH) and HF with preserved EF (HFpEF)-induced pulmonary hypertension or with HFpEF alone, serum relaxin-2 levels were significantly augmented in the group of patients with PAH (Table 2) [103]. In addition, the identification that relaxin-2 levels increase during worsening degrees of RV anomalies was predominantly observed in PAH settings, in which the occurrence of RV dysfunction is more common than in HFpEF [103,106]. The uncovering that serum relaxin-2 concentration is not increased in HFpEF patients is consonant with previous studies from shear stress and pressure models applied to pulmonary artery endothelial cells (a model of HFpEF), in which relaxin-2 levels remained unaltered [107]. In another study with chronic HFpEF patients, Emmens et al. (2017) could not determine an association between circulant relaxin-2 levels and pulmonary artery pressure (PAP) and right-sided heart function, although relaxin-2 plasma levels were detectable in all patients and showed higher levels (median 82.3 pg/mL) than previous studies (Table 2), suggesting an increase in circulant relaxin-2 levels during pulmonary hypertension [104]. Relaxin-2 is considered a functional antagonist of the vasoconstrictor effect of endothelin-1 due to its ability to inhibit endothelin-1 secretion when administered in a flow-chamber model of bovine pulmonary endothelial cells [70,108]. Moreover, it has also been reported that relaxin-2 is inversely correlated with endothelin-1 levels in patients with severe HF [70]. Taking into account the above-mentioned effects of relaxin-2 and given that endothelin-1 is one of the most potent vasoconstrictor neurohormonal factors involved in the pathophysiology of HF and PAH [109,110], and that endothelin-1 plasma levels are elevated during HF [100], it is conceivable that relaxin-2 is increased in PAH and HF with right-sided heart pressure overload as a compensatory protector mechanism against the pathologically increased activation of the vasoconstrictor mediators endothelin-1 and angiotensin (Ang) II [70,91,108]. 

Consequently, it is predicted that relaxin-2 could be a valuable biomarker for the identification of high-risk patients and direct clinical treatment, as well as for the evaluation of HF prognosis, representing a promising target for future therapeutic strategies [70,98]. Although it is widely accepted that relaxin-2 plays a role as a compensatory neurohumoral mediator in different settings of human HF, more studies will be needed to further understand the potential of this hormone as a biomarker of HF.

### 3.2. Atrial Fibrillation

Currently, some researchers have recognized that the pleiotropic hormone relaxin-2 could be effective for AF management for several reasons: (1) its distinguished antiarrhythmic and cardioprotective properties, like its anti-inflammatory, anti-apoptotic, anti-fibrotic, anti-hypertrophic, and anti-oxidant effects; (2) its capability to inhibit Ang II; and 3) its effect regulating ECM turnover and decreasing collagen excessive deposition in cardiac tissues [41,42,43,111,112]. Relaxin-2 achieves an outstanding anti-fibrotic effect through the regulation of cardiac fibroblast proliferation; the modulation of myofibroblasts differentiation and collagen synthesis; and the control of the expression of pro-fibrotic factors (including TGF-β1 or Ang II) and MMPs [41,42,43]. Several preclinical models have demonstrated that relaxin-2 reduces AF susceptibility after MI [111], and in aged hypertensive rats [112,113], being one of the possible mechanisms of the regulation of ionic currents and inotropy in cardiac cells [112,114]. Although only a limited number of reviews have addressed the effect of relaxin-2 in AF despite the numerous anti-fibrotic, anti-inflammatory, and anti-oxidant properties of this endogenous peptide at a cardiac level and its cardioprotective role demonstrated in preclinical models of AF and arrhythmic backgrounds [111,112,113,115,116,117], it is extremely relevant to highlight the role of relaxin-2 as a promising biomarker in AF.

AF patients have shown an increase in peripheral relaxin-2 levels when compared with patients with sinus rhythm, with higher levels in persistent AF than in paroxysmal AF (Table 3) [118]. Regarding the usefulness of relaxin-2 as a biomarker for risk stratification in the management of AF, serum relaxin-2 levels were higher in patients with than without AF recurrence, and elevated relaxin-2 serum levels were an independent predictor of post-radiofrequency catheter ablation (RFCA) AF recurrence (Table 3) [119]. Moreover, serum levels of relaxin-2 were positively correlated with an increase in left atrial diameter, with the risk of HF occurrence and with diverse serum fibrosis-related biomarkers such as TGF-β, procollagen type I C-terminal peptide (PICP), as well as TNF-α, demonstrating a close association between relaxin-2 and the progression of left atrial diameter as well as with fibrotic processes in AF [118]. 

For the above-mentioned reasons, relaxin-2 should be considered a potential predictor in AF that may help in the optimization and research of new treatment strategies. Future studies, and even clinical trials, are required to ascertain the specific implications of this hormone in the physiopathology of AF, which could be relevant for the diagnosis, risk stratification, management, and treatment of this disease.

### 3.3. Ischemic Heart Disease

At present, there exists evidence to sustain the hypothesis that the hormone relaxin-2 could counteract many physiopathologic mechanisms which take place in ischemic heart disease. Reperfusion therapy with relaxin-2 in several animal models of ischemia–reperfusion (I/R) injury or the preventive administration of this hormone before the induction of ischemia has shown promising cardioprotective results including the reduction of myocardial damage, cardiomyocyte contractile dysfunction, cardiomyocyte apoptosis, inflammatory leukocyte recruitment, platelet and mast cell activation, necrotic myocardial tissue area, and myocardial calcium content, together with an improvement of the ventricular performance and cardiac function and the stimulation of coronary vasodilatation [120,121,122,123]. Interestingly, these studies bring up the question of whether relaxin-2, in addition to its ability as a preventative drug that induces cardioprotection during I/R injury, could also be considered a useful biomarker for the prevention and diagnosis in advance of ischemic heart disease. 

It is hypothesized that endogenous relaxin-2 could act as a physiologic agent with a relevant role as a natural protector against cardiovascular disease [60,123]. This assumption, taken together with the fact that cardiomyocytes secrete relaxin-2 [124] and express RXFP1 [125,126], strongly suggests that relaxin-2 could be an endogenous cardiac agent implicated in the mechanisms of myocardial pre-conditioning. Clinical and epidemiological studies determined a reduction in the incidence of ischemic heart disease during ovulation in women, while in aged-matched men, menopause, or oral contraceptive use, the incidence of this syndrome is increased in parallel with the cessation of relaxin-2 luteal secretion, caused by the absence of ovarian activity [127]. Intriguingly, this assumption is in agreement with the work of Heringlake et al. (2009), who reported that patients suffering from ischemic heart disease and with severe myocardial dysfunction present lower levels of relaxin-2 in plasma than patients with preserved myocardial function (Table 4) and that plasmatic relaxin-2 concentration is inversely associated with the severity of heart disorders in these patients; these findings suggest that systemic relaxin-2 could act as a compensatory vasodilatory reaction hormone during ischemic heart disease [102]. Considering all this evidence, we would expect that endogenous relaxin-2 could be a relevant biomarker during clinical daily practice for the prevention of ischemic heart disease.

### 3.4. Myocardial Infarction

It has been extensively described that relaxin-2 induces a beneficial cardiac effect during the course of MI and exerts a protective effect in cardiac remodelling after MI. In particular, relaxin-2 reverts cardiac fibrosis (suppressing the protein expression levels of TGFβ1, α-SMA, and type I collagen), attenuates myocardial apoptosis, reduces cardiac inflammation (decreasing macrophage infiltration, mast cell degranulation, and the expression of inflammatory cytokines and chemokines), promotes vascular regeneration (increasing the expression of pro-angiogenic molecules and vessel density in the infarcted myocardium), ameliorates cardiac performance, decreases reperfusion-induced ventricular arrhythmias, preserves gap junction distribution, and ameliorates focal repolarization in the border zone of the infarcted region (through the reversion of connexin 43 disarrangement) in animal models of MI [111,115,135,136,137,138].

Regarding the myriad cardioprotective roles exerted by relaxin-2 in situations of I/R injury, along with the therapeutical and beneficial effects of this hormone during MI previously described, it is conceivable to propose that endogenous relaxin-2 may be considered a biomarker in this disease. However, little is known about the prognostic significance of relaxin-2 circulatory levels in MI patients. The only work studying serum levels of relaxin-2 during AMI reported that these levels were significantly higher in AMI patients compared with healthy individuals (Table 4), but no association was found between circulant relaxin-2 and other variables in the subjects of study [128]. These results might suggest an endogenous protective role of relaxin-2 during MI; however, additional large-scale prospective cohort studies are necessary to ascertain the potential causal link between this hormone and MI [92,128]. Recently, researchers in this field have declared that relaxin-2 may be considered a biomarker for predicting the probability of MI in the future and for the early diagnosis of this disease, which could guarantee instantaneous initiation of reperfusion therapy to potentially reduce the mortality rate during MI [139].

### 3.5. Aortic Valve Disease

There exists only one work that evaluated relaxin-2 circulatory levels in 60 patients with calcific aortic valve stenosis (CAVS), which showed that serum relaxin-2 concentrations were lower in patients with CAVS compared with healthy subjects (Table 4) [129]. Moreover, robust correlations were determined between relaxin-2 and several calcification markers in aortic valve leaflets from patients with CAVS: positive with elastin and negative with osteoprotegerin, osteocalcin, osteopontin, and valve calcification [129]. 

In this regard, the effect of relaxin-2 of inhibiting calcification and preserving elastin, together with its known roles in inhibiting vascular inflammation and the recruitment and adhesion of inflammatory cells to the endothelium, as well as mitigating chemokines, cytokines, and endothelial adhesion molecules expression at cardiac level, suggest the possible use of relaxin-2 as a biomarker and as a therapeutic strategy in situations of aortic valve stenosis [111,115,129,140,141,142]. However, there exists a necessity of further studies to unravel the specific influence of relaxin-2 on aortic valve disease, for example, determining the expression of relaxin-2 in the healthy valve or analysing the changes in circulatory relaxin-2 levels after aortic valve replacement [129]. Moreover, future works should evaluate relaxin-2 plasma levels in patients with CAVS-induced hypertension and with non-CAVS-related hypertension since this syndrome constitutes a known risk factor for CAVS and the calcification of the aortic valve could lead to the development of hypertension [143]. 

### 3.6. Hypertension

There exists a growing interest in the study of bioactive molecules, such as hormones or adipokines, as prognostic factors for the development of hypertension [144,145]. The hormone relaxin-2 is also considered an adipokine implicated in the protection against cardiometabolic diseases, being able to modulate inflammation, blood pressure and angiogenesis, induce vasodilatation, improve endothelial dysfunction during hypertension, and regulate fibrosis and ECM remodelling, two processes that predispose to hypertensive heart disease [146,147,148,149,150]. The myriad cardiovascular properties of relaxin-2 could potentially prevent some pathophysiological mechanisms that cause target organ damage during hypertension [92,151,152]. However, these possibilities notwithstanding, the specific role of endogenous relaxin-2 on hypertensive cardiovascular damage is not fully deciphered, although some authors have studied plasmatic levels of this hormone in patients with hypertension.

It has been described that relaxin-2 levels are significantly lower in patients with hypertension and/or with masked hypertension (frequently associated with higher risk for target organ damage, increased hazard for essential hypertension, and cardiovascular morbidity similar to hypertensive patients) compared with normotensives individuals (Table 4) [130,133]. During masked hypertension, relaxin-2 levels seems to be lower when compared with white coat hypertension (in which organ damage and cardiovascular events are less prevalent compared with essential hypertension) (Table 4) [131]. These findings suggest that a decrease in relaxin-2 plasma levels could contribute to the development of essential hypertension, taking into account that white coat hypertension is less harmful than masked hypertension [131,153]. Moreover, relaxin-2 plasma levels are negatively correlated to brachial and central aortic diastolic and systolic pressures in hypertensive patients [130], which could be interesting for the prediction of target organ damage in those patients [154]. 

Curiously, circulatory levels of relaxin-2 are lower in the young healthy offspring of hypertensive patients compared with the young healthy offspring of normotensive patients in the absence of major cardiovascular risk factors (Table 4) [132]. In situations of PAH, relaxin-2 plasma levels are augmented when compared with healthy conditions, and these levels are positively correlated with pulmonary vascular resistance (PVR) and the degree of RV dysfunction (Table 4) [103]. These results suggest that relaxin-2, a well-known anti-inflammatory and anti-fibrotic hormone, is upregulated as a compensatory mechanism to counteract the inflammation and fibrosis that persist during PAH settings, as previously described [71,103,155]. The concept of this compensatory increase in the hormone relaxin-2 in response to PAH and RV anomalies was also supported by clinical trials in which serelaxin administration to AHF patients induced a vasodilator effect on the pulmonary vessels, causing a notable decrease in pulmonary capillary wedge pressure (PCWP), PAP, PVR, and right atrial (RA) pressure [156].

Considering this evidence, relaxin-2 levels could be employed as a promising biomarker for the early detection of essential hypertension and concurrent cardiovascular events, and for categorizing children and young adults according to their cardiovascular risk as a basis for the prevention of adult cardiac disorders. Nevertheless, additional studies are required to completely clarify if endogenous relaxin-2 is related to hypertensive cardiovascular damage.

### 3.7. Atherosclerosis

The endogenous hormone relaxin-2 possesses a vascular gelatinase activity with the ability to upregulate the synthesis of MMP-2 and -9 in arteries, contributing to vasodilatation, hyperfiltration, and reduction of myogenic reactivity through the activation of the endothelial endothelin (ET)_B_ receptor-nitric oxide (NO) vasodilatory pathway [157,158,159]. In fact, MMPs and TIMPs are responsible for the ECM degradation and vascular remodelling which takes place during vascular diseases [160]. Furthermore, the beneficial effects of relaxin-2 in atherosclerosis also comprise the stimulation of vasculogenesis via the upregulation of vascular endothelial growth factor (VEGF) transcript [161,162], the prevention of endothelial dysfunction, the reduction of systemic arterial resistance, the increase of global artery compliance [148,163,164], the amelioration of increased responsiveness to Ang II [165], and the inhibition of atherosclerotic plaque development [166]. Interestingly, relaxin-2 exerts an anti-inflammatory effect by decreasing IL-6 mRNA expression and plasma levels in rat left ventricle and infarcted myocardium as well as in apolipoprotein E-deficient mice model of atherosclerosis [111,115,166]. These outcomes could be interesting in the context of atherosclerosis because IL-6 is considered a pro-atherogenic cytokine [167]. Moreover, clinical studies have described that IL-6 serum levels are elevated during unstable angina and are considered an independent risk factor for coronary artery disease, suggesting the use of IL-6 as a biomarker for evaluating the inflammatory response and severity of the disarrangements arising from atherosclerosis [168,169]. In this context, it is conceivable to hypothesize that relaxin-2 may be involved in several vascular diseases such as atherosclerosis, despite the lack of recent reports deciphering the specific role of relaxin-2 during the atherosclerotic process [133,166,170].

Serum levels of relaxin-2 are higher in the early stages of atherosclerosis and gradually decrease with the progression of the disease, eventually showing comparable results between patients with permanent ischemic manifestations from target organs and healthy subjects (Table 4) [134]. Supporting these findings, an inverse association of arterial tissue relaxin-2 mRNA levels with the clinical severity of atherosclerotic lesions was recently reported [170]. Tissue relaxin-2 mRNA levels are inversely correlated with MMP-2 in mild atherosclerosis and positively correlated with eNOS in moderate atherosclerosis, which supports the beneficial effect of relaxin-2 in this pathology [170]. Recently, Klimontov et al. (2022) found no associations between serum levels of relaxin-2 and carotid atherosclerosis in patients with type 2 diabetes (T2D) [171]. A feasible explanation of these results could be that some confounders, such as insulin sensitivity, may modify the relationship between relaxin-2 and atherosclerosis in diabetes [171]. 

Those insights could indicate that relaxin-2 acts as a compensatory response during the reduction in blood supply in the early stages of atherosclerosis, a mechanism that presumably becomes ameliorated in the late clinical stages of the disease [134]. Despite this, additional mechanistic insights on the underlying processes of this effect need to be addressed, and further studies with consistent and bigger populations are required to determine the possible clinical use of relaxin-2 as a biomarker in atherosclerotic backgrounds.

## 4. Limitations and Future Perspectives

As has been previously described in the literature, the concentration of relaxin-2 in plasma and/or serum is highly variable depending on the physiopathological conditions of the population of study, which provide heterogeneous results that could generate uncertainty about the role of the hormone relaxin-2 as a biomarker. Moreover, it should be pointed out that there exists a profound disagreement regarding the validation, accuracy, sensibility, and reproducibility of the commercially available human relaxin-2 ELISA assay kits, which usually are not able to determine low concentrations of relaxin-2 and could generate doubt regarding the interpretation of the data [172]. Different reasons could explain these discrepancies: (a) highly heterogeneous study populations regarding gender, age, CVD presentation and aetiology, and LVEF; (b) a high variability in relaxin-2 circulating levels in both groups of study, small sample sizes of the groups, which decreased statistical power; and (c) employment of different antiserum/immunoassays batches or with different detection limits [91]. For this reason, a relevant practice should be the repetition of the relaxin-2 determination but choosing the most suitable and reliably commercial human relaxin-2 ELISA kits [172]. Variability in relaxin-2 levels could be also caused by different preprocessing methodologies performed in the studies. For instance, some investigators do not use protease inhibitors (such as aprotinin) that avoid peptide degradation during blood sampling, a fact that may explain lower or indetectable circulatory relaxin-2 levels in certain studies [91,133]. However, the presence of determinations below the detection limit of the ELISA kits is sometimes unavoidable because some individuals have insignificant relaxin-2 concentrations, which is common in healthy conditions different from pregnancy, where relaxin-2 functions as a paracrine or autocrine factor to induce multiple physiological roles [67,70,71,172].

Even though several works have analysed the concentrations of relaxin-2 in plasma and/or serum in patients with established CVDs, most of them have not studied in depth the possible relationship between relaxin-2 circulating levels and different risk and/or prognostic markers related to the metabolic state and body composition [91,99,118,119,173]. Consequently, providing a strong foundation for this promising field of research could aid in implementing the possible use of relaxin-2 during daily clinical practice as a biomarker, risk predictor, or therapeutic agent in several CVDs. These outcomes could be reached through the cooperation of basic and clinical researchers in the design of, for example, clinical trials that might provide new insights about the function of relaxin-2 and its possible compensatory role in different backgrounds of cardiovascular pathology. 

## 5. Conclusions

Although recent preclinical and clinical outcomes have encouraged the potential use of relaxin-2 as a therapeutic strategy in patients suffering from different cardiovascular disarrangements owing to the myriad cardioprotective effects of this natural molecule, the scientific community cannot disregard the promising implications of endogenous relaxin-2 as an excellent biomarker in cardiovascular health and disease.

Relaxin-2 circulatory levels are clearly modified during cardiovascular damage when compared with healthy states (apart from pregnancy), where relaxin-2 levels remain low or even undetectable. It has been described that during the presence of CVDs (such as HF, AF, ischemic heart disease, MI, aortic valve disease, hypertension, and atherosclerosis) endogenous relaxin-2 levels suffer significant variations. However, at present, the physiopathological mechanisms that modulate these changes during events of cardiovascular disarrangements have not been clearly unravelled and characterized.

Deciphering relaxin-2’s biological and physiopathological role in distinct settings of CVDs will foster its clinical use as a therapeutic target and probably as a circulating biomarker in the near future, aiding in developing a personalized patient-based approach and leading specific therapies to particular patient groups with different CVDs. Moreover, assaying relaxin-2 systemic levels could be used as a relevant routine daily clinical tool as a predictor for the diagnosis, risk stratification, or management of several cardiovascular syndromes.

## Figures and Tables

**Table 2:** Relaxin-2 plasma or serum levels in different settings of heart failure.

			Heart Failure			
Condition	Patients (n)	Controls (n)	Relaxin-2 Levels in Controls	Relaxin-2 Levels in Patients	Main Results	Reference
CHF	Severe CHF-NHYA class IV (9♂:5♀)	Subjects undergoing cardiac catheterization and with no structural CVD (9♂:4♀)	<5 pg/mL	18.9 ± 3.4 pg/mL	↑ Relaxin-2 plasma levels in patients ↑ Relaxin-2 synthesis by failing myocardium	[70]
	Moderate CHF-NHYA class II (9♂:4♀)			7.9 ± 1.2 pg/mL		
CHF	Chronic HFrEF (51♂:36♀)	No controls	<2 pg/mL considered normal	89 pg/mL	↑ Relaxin-2 levels when compared with controls No relation between relaxin-2 and outcome	[97]
CHF	Chronic HFrEF-NYHA classes II to III (35)	Healthy subjects (10)	Rest: 19 ± 13 pg/mL	Rest: 72 ± 177 pg/mL	Relaxin-2 plasma levels did not discriminate between patients with and without CHF	[101]
			Peak exercise: 31 ± 31 pg/mL	Peak exercise: 59 ± 125 pg/mL		
AVS and HF	AVS free of HF (88)	Subjects undergoing invasive electrophysiologic study (6♂:5♀)	42 pg/mL	32 pg/mL	No changes in relaxin-2 plasma levels between groups Relaxin-2 secretion by the failing and extraction by the non-failing myocardium	[100]
	AVS with HF (41)			28 pg/mL		
	AVS with HFpEF (25)			40 pg/mL		
	AVS with HFrEF (16)			18 pg/mL		
IHD and HF	HF secondary to IHD (35♂:5♀)	No controls	-	20 pg/mL	↓ Relaxin-2 levels during recovery and positively correlated with cardiac output and inversely correlated with natriuretic peptides	[102]
HFpEF and PAH	HFpEF (14)	Subjects free of significant cardiovascular or systemic disease (18)	13.4 pg/mL *	12.9 pg/mL *	↑ Relaxin-2 levels in patients with PAH	[103]
	HFpEF-PAH (33)			12.8 pg/mL *		
	PAH (31)			46.6 pg/mL *		
CHF	CHF-NYHA classes I to IV (81♂:34♀)	Patients without CHF (20♂:11♀)	0.390 pg/mL	0.593 pg/mL	↑ Relaxin-2 plasma levels in patients Relaxin-2 can predict severe cardiovascular events in CHF within 180 days after discharge	[98]
PPCM	First quartile (0–11 days) (25♀)	No controls	-	97 ± 203 pg/mL *	↑ Relaxin-2 levels are associated with faster myocardial recovery	[90]
	Second quartile (12–24 days) (27♀)			10 ± 31 pg/mL *		
	Third quartile (25–51 days) (25♀)			4 ± 2 pg/mL *		
	Fourth quartile (52–95 days) (23♀)			5 ± 7 pg/mL *		
CHF	81 decompensated CHF (41♂:40♀)	36 (16♂:20♀)	36.7 pg/mL	67.1 pg/mL	↑ Relaxin-2 levels in patients Nonlinear correlation between relaxin-2 and NYHA cardiac function	[99]
HFpEF and PH	HFpEF and PH (15♂:36♀)	No controls	-	82.3 pg/mL	No correlation between relaxin-2 levels and PAP and right heart overload	[104]
AHF	AHF-HFrEF (74)	No controls	-	33.0 pg/mL *	Relaxin-2 levels are associated with right heart overload and pulmonary hypertension	[91]
	AHF-HFpEF (43)			28.5 pg/mL *		

Table 2 summarizes the most relevant works published until now describing relaxin-2 circulatory levels in different backgrounds of heart failure (HF). Relaxin-2 concentration is expressed in mean ± standard deviation (SD), median or median (interquartile range (IQR)) depending on the study. * Measured in serum. ↑: relaxin-2 levels increase. ↓: relaxin-2 levels decreased. AVS: aortic valve stenosis. CHF: chronic heart failure. CVD: cardiovascular disease. HF: heart failure. HFpEF: heart failure with preserved ejection fraction. HFrEF: heart failure with reduced ejection fraction. IHD: ischemic heart disease. NHYA: New York Heart Association. PAH: pulmonary arterial hypertension. PAP: pulmonary artery pressure. PH: pulmonary hypertension. PPCM: peripartum cardiomyopathy.

**Table 3:** Relaxin-2 plasma or serum levels during atrial fibrillation.

Atrial Fibrillation						
Condition	Patients (n)	Controls (n)	Relaxin-2 Levels in Controls	Relaxin-2 Levels in Patients	Main Results	Reference
AF	Paroxysmal AF (46♂:34♀)	Patients with sinus rhythm (75♂:41♀)	170.21 ± 85.45 ng/L *	244.95 ± 83.55 ng/L *	↑ Relaxin-2 levels with the development of AF Relaxin-2 is associated with fibrosis-related biomarkers	[118]
	Persistent AF (73♂:42♀)			269.47 ± 77.24 ng/L *		
AF	Patients with AF recurrence (33♂:20♀)	No controls	-	382.21 ± 149.89 ng/L *	↑ Relaxin-2 level in patients with than without AF recurrence	[119]
	Patients without AF recurrence (124♂:71♀)			275.42 ± 108.70 ng/L *		
	Paroxysmal AF (83♂:44♀)			293.51 ± 124.06 ng/L *		
	Persistent AF (76♂:45♀)			303.21 ± 128.80 ng/L *		

Table 3 summarizes the works describing relaxin-2 circulatory levels during atrial fibrillation (AF). Relaxin-2 concentration is expressed in mean ± standard deviation (SD) or median (interquartile range (IQR)) depending on the study. * Measured in serum. ↑: relaxin-2 levels increase. ↓: relaxin-2 levels decreased. AF: atrial fibrillation.

**Table 4:** Relaxin-2 plasma or serum levels during ischemic heart disease, myocardial infarction (MI), aortic valve disease, hypertension, and atherosclerosis.

Ischemic Heart Disease						
Condition	Patients (n)	Controls (n)	Relaxin-2 Levels in Controls	Relaxin-2 Levels in Patients	Main Results	Reference
IHD with HF	IHD with high degree and low degree of HF (35♂:5♀)	No controls	-	20 pg/mL	↓ Relaxin-2 levels in patients with IHD than patients with preserved myocardial function	[102]
**Myocardial Infarction**						
AMI	Patients who presented AMI for the first time (63♂:17♀)	Healthy subjects (61♂:19♀)	9.2 ± 2.3 ng/mL *	27.4 ± 6.3 ng/mL *	↑ Relaxin-2 levels in AMI patients compared with controls	[128]
**Aortic Valve Disease**						
CAVS	Patients scheduled to undergo surgery for severe CAVS (60)	Healthy subjects (20)	0.5 ± 0.08 ng/mL *	0.02 ± 0.005 ng/mL *	↓ Relaxin-2 levels in patients with CAVS compared with controls Relaxin-2 levels are correlated with several calcification markers	[129]
**Hypertension**						
HT	Never treated patients with HT (40)	Normotensive individuals (42)	49.7 ± 39.8 pg/mL *	36.5 ± 7.3 pg/mL *	↓ Relaxin-2 levels in HT compared with controls	[130]
Masked HT and white coat HT	Masked HT	No controls	-	35.2 ± 6.7 pg/mL	↓ Relaxin-2 levels in patients with masked HT	[131]
	White coat HT			46.8 ± 23.6 pg/mL		
	Arterial aneurysm (16♂)			49.39 ± 8.62 pg/mL *		
	Patients undergoing TAB (4♂:2♀)			15.86 ± 4.29 pg/mL *		
Healthy offspring of patients with essential HT	Healthy offspring of HT patients (24♂:22♀)	Healthy offspring of healthy parents (28♂:22♀)	10 ± 5 pg/mL	6 ± 3 pg/mL	↓ Relaxin-2 levels in healthy offspring of HT parents	[132]
Masked HT	Patients with masked HT (8♂:16♀)	Healthy normotensives (52♂:54♀)	56.8 ± 13.6 pg/mL	35.2 ± 6.7 pg/mL	↓ Relaxin-2 levels in masked HT compared with controls	[133]
PAH and HFpEF	PAH (31)	Subjects free of significant cardiovascular or systemic disease (18)	13.4 pg/mL *	46.6 pg/mL *	↑ Relaxin-2 levels in PAH compared with healthy controls Relaxin-2 positively associated with PVR and RV dysfunction	[103]
	HFpEF-PAH (33)			12.8 pg/mL *		
	HFpEF (14)			12.9 pg/mL *		
			**Atherosclerosis**			
ATH and arterial aneurysm	ATH patients (16♂:5♀)	Healthy subjects (7♂:3♀)	10.32 ± 1.35 pg/mL *	16.22 ± 4.70 pg/mL *	↑ Relaxin-2 levels in arterial aneurysm patients ↑ Relaxin-2 levels at early stages of ATH	[134]
	Arterial aneurysm (16♂)			49.39 ± 8.62 pg/mL *		
	Patients undergoing TAB (4♂:2♀)			15.86 ± 4.29 pg/mL *		

Table 4 summarizes the works published until now describing relaxin-2 circulatory levels during ischemic heart disease, myocardial infarction (MI), aortic valve disease, hypertension and atherosclerosis. Relaxin-2 concentration is expressed in mean ± standard deviation (SD) or median (interquartile range (IQR)) depending on the study. * Measured in serum. ↑: relaxin-2 levels increase. ↓: relaxin-2 levels decreased. AMI: acute myocardial infarction. ATH: atherosclerotic disease. CAVS: calcific aortic valve stenosis. HF: heart failure. HFpEF: heart failure with preserved ejection fraction. HT: hypertension. IHD: ischemic heart disease. PAH: pulmonary arterial hypertension. PVR: pulmonary vascular resistance. RV: right ventricle. TAB: temporal artery biopsy.

## Data Availability

Not applicable.
